# The complete mitochondrial genome of the Korean endemic earthworm *Amynthas susakii* Kobayashi 1936 (Clitellata: megascolecidae)

**DOI:** 10.1080/23802359.2025.2569554

**Published:** 2025-10-08

**Authors:** Jachoon Koo, Yong Hong

**Affiliations:** a Division of Science Education and Institute of Fusion Science, College of Education, Jeonbuk National University, Jeonju, Korea; b Department of Plant Medicine, College of Agriculture & Life Sciences, Jeonbuk National University, Jeonju, Republic of Korea

**Keywords:** Megascolecidae, *Amynthas susakii*, mitochondrial DNA, molecular phylogeny

## Abstract

*Amynthas susakii* (Kobayashi 1936) is an earthworm species endemic to Korea that typically inhabits mountainous forest environments. The specimens were collected from Mount Jiri, South Korea. The complete mitogenome of *A. susakii* was sequenced, assembled, and annotated. The mitogenome of *A. susakii* is a circular DNA molecule of 15,115 bp with an A + T content of 67.7%, comprising 13 protein-coding genes, 2 ribosomal RNA genes, 22 transfer RNA genes, and 1 control region. Phylogenetic analysis showed that *A. susakii* clustered with other *Amynthas* species in the well-supported Megascolecidae family.

## Introduction

Some species of the genus *Amynthas* Kinberg (1867) have been transported by humans and other organisms to various parts of the world, including the Neotropical and Nearctic regions (Gates 1972; Sims and Easton 1972). The species of the *Amynthas* group have a wide range of ecological requirements and occupy a wide range of regions, soil types, and vegetation worldwide. However, certain species are found in limited areas. The species examined in this study exhibited a particular set of ecological characteristics. *Amynthas susakii* (Kobayashi, 1936) is mainly found in natural forests.

The complete mitochondrial genomes (mitogenomes) of only 29 species of the family Megascolecidae are available (Boore and Brown 1995; Wang et al. 2015; Zhang et al. 2016a, 2016b; Hong et al. 2017; Zhang et al. 2019; Kim and Hong 2022; Koo and Hong 2023, 2025). Most of these mitogenomes belong to the *Amynthas* group*. Amynthas* contains more species than any other genus in the *Pheretima* complex group (Sims and Easton 1972). The *Amynthas* group is heterogeneous and encompasses a wide range of species, including some species in the genus *Metaphire*. It includes species with fasciculate and pinnate longitudinal musculature (Csuzdi and Zicsi 2003); a characteristic that is highly significant in earthworm phylogeny (Pop 1941; Omodeo 1956; Zicsi 1985). However, previous mitogenomic analyses have demonstrated that this characteristic exhibits a high degree of homoplasy (Shekhovtsov et al. 2020; Csuzdi et al. 2022) and consequently, provides limited value for resolving higher-level taxonomic relationships.

The mitogenomic sequences of *Amynthas* provide valuable information for constructing mitogenome phylogenies and advancing our understanding of Clitellata mitogenomic evolution (Koo and Hong 2023). However, many species are yet to be analyzed.

## Materials and methods

**Figure 1.**
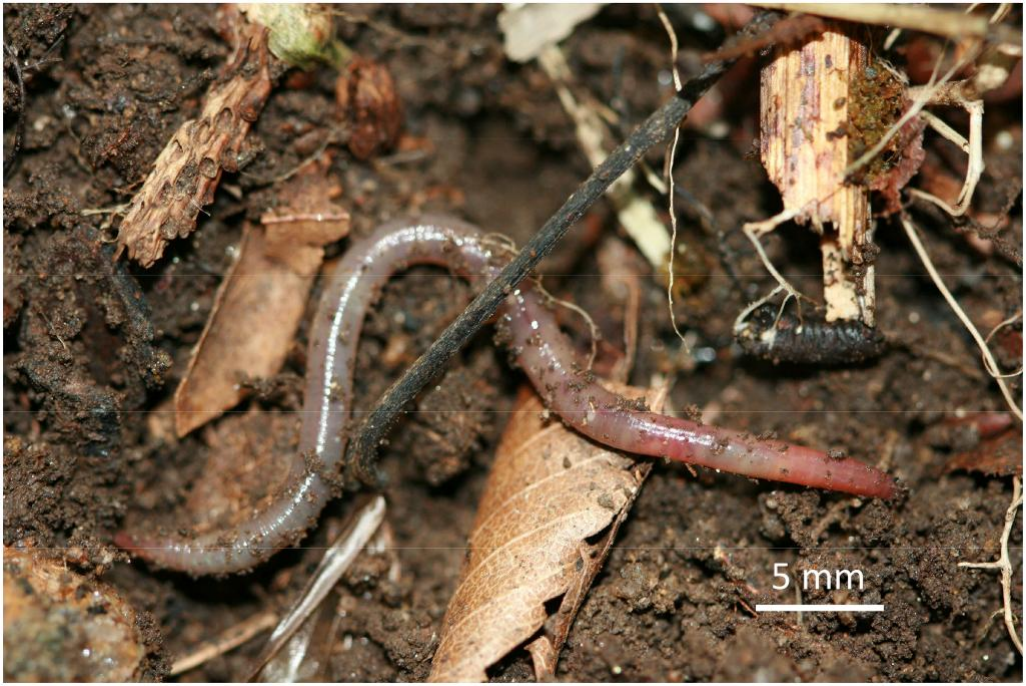
Reference images of *Amynthas susakii* collected from Mt. Jiri in korea. Images were captured using a canon digital camera by yong hong on 31 july 2011. Scale bars: 5 mm.

Specimens of *A. susakii* were collected from Mt. Deogyu, Jeollabuk-do, South Korea (35°52′12″N, 127°48′49″E; 1,296 m elevation) on August 5, 2020. The material was obtained by manually sorting litter layers and soil from a mountainous forest. *Amynthas susakii* is a small, brownish earthworm, measuring 44–64 mm in length and approximately 3.0 mm in diameter, with 94–106 segments (Figure 1; Kobayashi 1936). It possesses two pairs of spermathecal pores located at intersegments 6/7 and 7/8, along with distinct genital papillae.

All examinations were conducted through external observation of whole specimens and dorsal dissection under a stereomicroscope (Zeiss KCX-160). A voucher specimen was deposited at Jeonbuk National University, Jeonju, South Korea, under accession number JBNU0006 (contact: Yong Hong, yonghong@jbnu.ac.kr).

### DNA extraction and mitogenome sequencing, assembly, and annotation

Total genomic DNA was extracted from body segments of a single *A. susakii* individual using the DNeasy Blood & Tissue Kit (Qiagen, USA) following the manufacturer’s protocol. The extracted DNA was sheared and size-selected for a target insert size of ∼350–450 bp, followed by paired-end sequencing with 150 bp reads on the Illumina HiSeq X platform. Sequencing adapters were ligated to both ends of the fragmented DNA using the TruSeq DNA Nano Library Prep Kit (Illumina Inc., USA) to construct a paired-end sequencing library. High-throughput sequencing was performed using an Illumina HiSeq X platform, yielding 44,314,708 raw reads. The raw reads were processed using Trimmomatic v0.38 to ensure data quality (Bolger et al. 2014). Adapter sequences were removed, and bases with a Phred quality score <3 were trimmed from the read ends. Reads <36 bp were discarded, resulting in 33,664,872 high-quality reads. Filtered reads were assembled using a two-pass reference-assisted strategy to recover the complete circular mitogenome. First, we performed broad de novo assembly and identified a mitochondrial contig by comparison with the *Amynthas* reference mitogenome (GenBank OL321943). All filtered reads were then mapped to this contig/reference, and only the mapped read pairs (59,254 reads) were re-assembled with SPAdes v3.13.0, yielding a single 15,115-bp circular mitogenome (Bankevich et al. 2012). Circularization and accuracy were supported by self-mapping the reads to the final mitogenome using Bowtie2 in Geneious Prime (100% breadth; mean depth ∼471×; Supplementary Figure S1).

The complete circular mitochondrial genome was annotated using the MITOS2 web server (http://mitos2.bioinf.uni-leipzig.de/; Donath et al. 2019). To ensure the accuracy of the gene boundary assignments, the initially annotated gene positions were manually verified and refined using BLAST searches against the NCBI nucleotide database. A graphical representation of the mitochondrial genome was constructed using the Proksee web platform (https://proksee.ca/; Grant et al. 2023) employing the relative-scale visualization option. Nucleotide compositional bias was assessed using the following formulas: AT-skew = (A − T)/(A + T) and GC-skew = (G − C)/(G + C), as described by Perna and Kocher.

### Phylogenetic analysis

Phylogenetic analysis was conducted using maximum likelihood (ML) with IQ-Tree v2.2.0 (Nguyen et al. 2015), based on the nucleotide sequences of 13 mitochondrial protein-coding genes (PCGs). The mitogenome dataset included publicly available complete mitogenome sequences of 31 Megascolecidae species and 1 representative lumbricid species, *Lumbricus terrestris,* as the outgroup. This species is widely used in phylogenetic analyses and is the first terrestrial earthworm group to undergo complete mtDNA analysis (Boore and Brown 1995). Each PCG was individually aligned using MAFFT (Katoh and Standley 2013), and poorly aligned regions or gap-rich sites were eliminated using trimAl (Capella-Gutiérrez et al. 2009). Default parameters were applied for both the alignment and trimming procedures. The resulting alignments were concatenated into a single dataset, which was used to reconstruct a phylogenetic tree using the ML method. ModelFinder implemented in IQ-TREE was used to identify the best-fit substitution models for each partition using the Bayesian information criterion to determine the most appropriate substitution models for phylogenetic analysis. The following partitioning schemes and corresponding models were selected as optimal: TIM2 + F + R5 for COX1 + COX2 + COX3 + CYTB+ATP6 + ND1, TPM3 + F + R4 for ATP8 + ND6 + ND2, and GTR+F + R5 for ND5 + ND4L + ND4 + ND3. The ML phylogeny was inferred using 5000 ultrafast bootstraps (Minh et al. 2020).

## Results

### Complete mitochondrial genome structure of A. susakii

**Figure 2.**
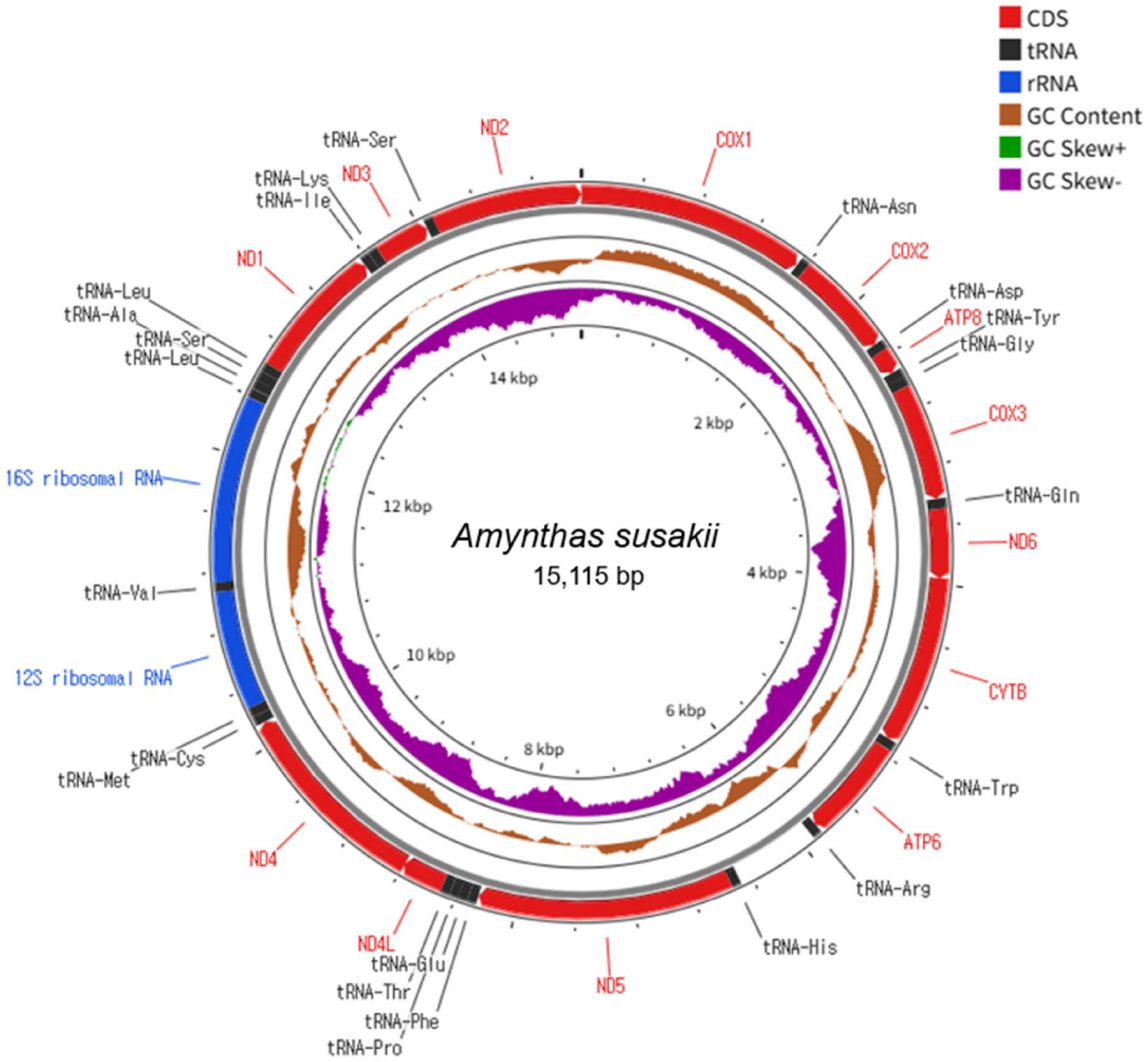
Mitochondrial map of *amynthas susakii*. Circular maps were generated with the proksee web server using the relative scale option. Protein-coding genes are shown as red, rRNA genes as blue, and tRNA genes as black. Inner rings display base-composition metrics: GC content is plotted as the deviation from the genome-wide mean (mean GC = 32.29%). GC-skew is plotted as absolute values of (G−C)/(G + C) in 500-bp windows (step = 1 bp); the genome-wide mean GC-skew is ∼−0.165.

The complete mitochondrial genome of *A. susakii* is a circular DNA molecule of 15,115 bp (GenBank accession no. PV631717) comprising 37 genes typical of earthworm mitogenomes (Figure 2). These included 13 PCGs, 22 transfer RNA genes, 2 ribosomal RNA genes, and a control region. This gene arrangement was consistent with that observed in other megascolecid species.

The overall nucleotide composition of the mitogenome was 34.7% adenine (A), 33.0% thymine (T), 13.5% guanine (G), and 18.8% cytosine (C), indicating a marked A + T bias. The total A + T content was 67.7%. Among all PCGs, *ND6* exhibited the highest A + T content (71.6%).

Compositional asymmetry was further assessed using AT-skew and GC-skew, with values calculated at 0.0259 and −0.1626, respectively. All 13 PCGs initiated translation using the conventional ATG start codon. Regarding termination, the majority of PCGs utilized canonical stop codons, such as TAA (*COX1*, *CYTB*, *ATP6*, *ND4L*, and *ND3*) and TAG (*COX2* and *ND1*), whereas others ended with incomplete stop codons (a single T), as observed in *ATP8*, *COX3*, *ND6*, *ND5*, *ND4*, and *ND2*.

### Genetic relationship of the genus amynthas

**Figure 3.**
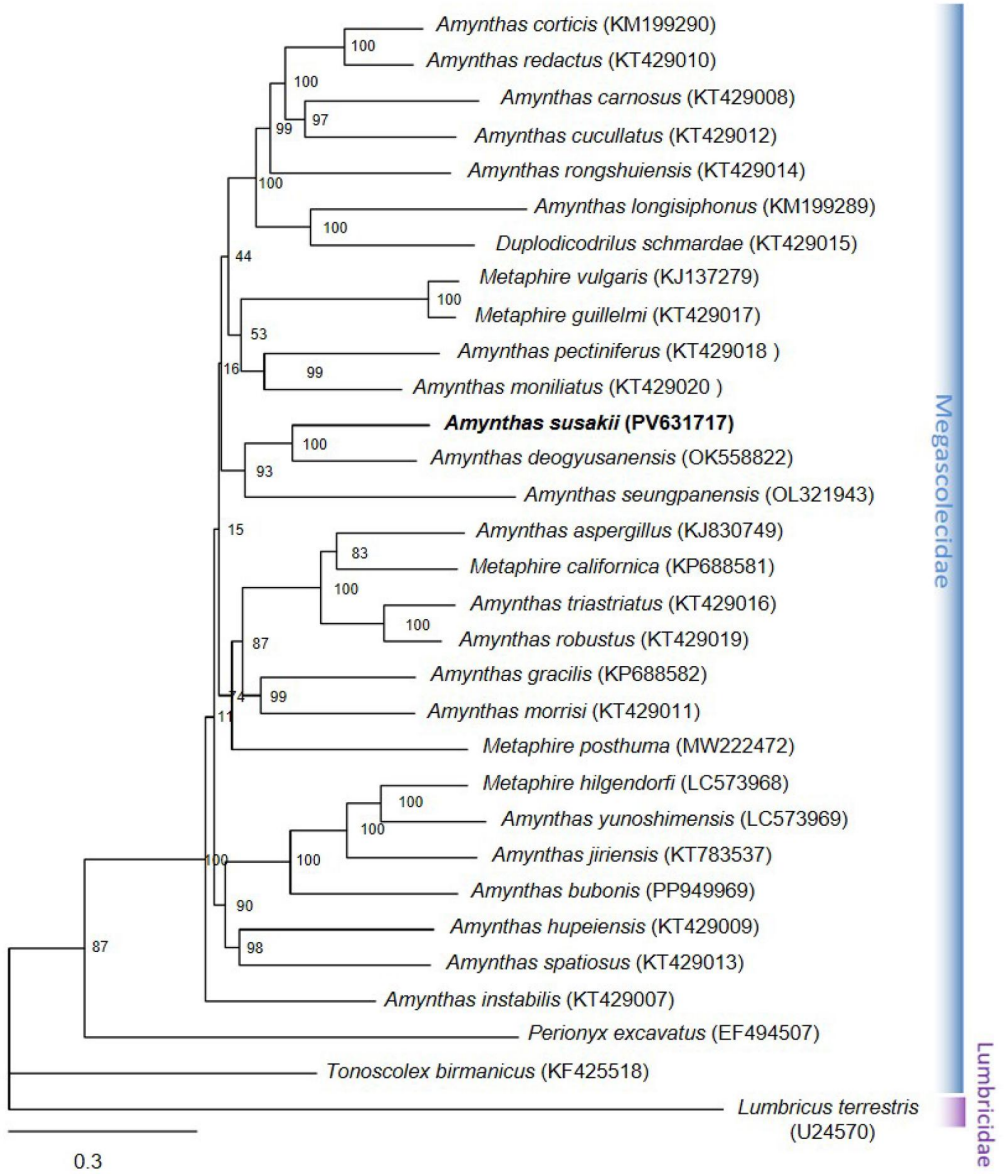
Phylogenetic relationships among 29 megascolecidae species based on the nucleotide sequences of 13 PCGs in the mitogenome. *Lumbricus terrestris* was used as an outgroup. Phylogenetic trees were constructed using maximum likelihood (ML) methods. Numbers on nodes indicate ML bootstrap values (%). the GenBank accession numbers were marked after the species name. The following sequences were used: *Amynthas aspergillus* (Zhang et al. 2016a), *amynthas carnosus, amynthas cucullatus, amynthas hupeiensis, amynthas instabilis, amynthas morrisi, amynthas moniliatus, amynthas pectiniferus, amynthas redactus, amynthas robustus, amynthas rongshuiensis, amynthas spatiosus, amynthas triastriatus, metaphire guillelmi, duplodicodrilus schmardae* (Zhang et al. 2016b), *amynthas corticis, amynthas gracilis, amynthas longisiphonus, metaphire californica, amynthas jiriensis* (Hong et al. 2017), *amynthas seungpanensis* (Kim and Hong 2022), *metaphire hilgendorfi, amynthas yunoshimensis* (seto et al. 2021), *amynthas deogyusanensis* (Koo and Hong 2023), *amynthas bubonis* (Koo and Hong 2025), *amynthas susakii* (this study), *metaphire vulgaris*, *perionyx excavatus* (unpublished), *tonoscolex birmanicus* (Wang et al. 2015), and *lumbricus terrestris* (Boore and Brown 1995).

A ML phylogenetic tree was constructed using concatenated nucleotide sequences of 13 mitochondrial PCGs from 31 earthworm species. In the resulting tree topology, *A. susakii* nested within the family Megascolecidae, forming a well-supported monophyletic clade with *Amynthas deogyusanensis* (bootstrap support = 100) and *Amynthas seungpanensis* (bootstrap support = 94) (Figure 3). This clade highlighted the evolutionary relationships among *Amynthas* species and indicated that *A. susakii* shares a recent common ancestor with these congeners. The branching pattern suggested a strong phylogenetic affinity and close evolutionary proximity among these three species within Megascolecidae.

*Amynthas susakii* was most closely related to *Amynthas deogyusanensis*, which shares a high degree of sequence similarity and evolutionary affinity, suggesting a relatively recent divergence or potential taxonomic closeness. This phylogenetic placement aligned well with morphological similarities and overlapping geographic distributions in East Asia. Overall, the positioning of *A. susakii* provides meaningful insights into the evolutionary relationships within the family Megascolecidae and reinforces its classification within the core *Amynthas* lineage.

## Discussion and conclusion

The complete mitogenome reported here is expected to provide a valuable resource for future studies on *A. susakii* and clarify the taxonomic status of the family Megascolecidae. Further analyses incorporating additional species from this group, as well as related genera, such as *Pheretima* and *Pithemera*, will be instrumental in elucidating the evolutionary relationships among the higher taxa of terrestrial earthworms. However, information on Megascolecidae mitogenomes remains scarce. Expanding the available mitogenomic data for Oligochaeta species beyond Megascolecidae will facilitate more robust phylogenetic analyses based on mitogenomic evidence (Boore and Brown 1995). In particular, a more comprehensive sampling of the diverse genera within this clade is needed to refine our understanding of their evolutionary history.

The monophyletic grouping of *A. susakii*, *A. deogyusanensis*, and *A. seungpanensis* collected from South Korea likely reflects a shared biogeographic origin and potential historical gene flow. As members of the family Megascolecidae, these species may have diverged relatively recently from a common ancestor that adapted to the ecological conditions of the Korean Peninsula. Their co-occurrence in similar temperate forest habitats suggests that exposure to comparable environmental pressures could limit genetic divergence. Furthermore, the shared biogeographic context may have promoted dispersal and interbreeding among ancestral populations, contributing to the high genetic similarity observed in mitochondrial phylogenetic analyses.

## Supplementary Material

Supplementary data_0915.docx

## Data Availability

The genome sequence data supporting the findings of this study are publicly available under GenBank accession no. PV631717. The associated BioProject, SRA, and Bio-Sample numbers are PRJNA769829, SRR33821090, and SAMN48884673, respectively.
