# Supplementary material for: The complete mitochondrial genome of the Korean endemic earthworm Amynthas susakii Kobayashi 1936 (Clitellata: megascolecidae)
Source: Mitochondrial DNA B Resour. 2025 Oct 8;10(11):1026–31. doi: 10.1080/23802359.2025.2569554 (PMC12509296; doi:10.1080/23802359.2025.2569554)
Supplement: Supplementary data_0915.docx [file TMDN_A_2569554_SM4878.docx]

**Supplementary data**

**The complete mitochondrial genome of the Korean endemic earthworm *Amynthas susakii* Kobayashi, 1936 (Clitellata: Megascolecidae)**

Jachoon Koo^1^, and Yong Hong^2^

^1^Division of Science Education and Institute of Fusion Science, College of Education, Jeonbuk National University, Jeonju 54896, Korea; [jkoo@jbnu.ac.kr](mailto:jkoo@jbnu.ac.kr) https://orcid.org/ 0000-0002-3559-326X

^2^Department of Plant Medicine, College of Agriculture & Life Sciences, Jeonbuk National University, Jeonju 54896, Republic of Korea; yonghong@jbnu.ac.kr <https://orcid.org/0000-0002--8093-9717>

^2^ Correspondence:

Yong Hong

Tel: +82-63-270-2529, Fax: +82-63-270-2531, e-mail: yonghong@jbnu.ac.kr

**
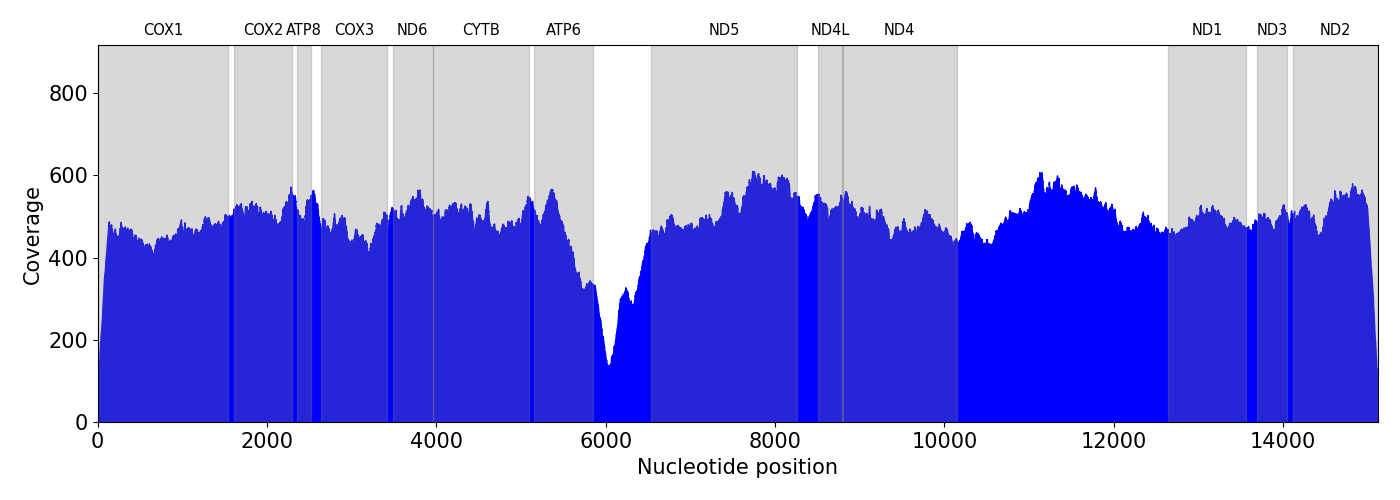
**

**Supplementary Figure S1.** **Genome-wide read coverage profile of the** Amynthas susakii **mitogenome.** Sequencing reads were aligned to the A. susakii mitochondrial genome using Bowtie2, and the resulting BAM file was processed with Samtools to compute per-base coverage depth. Read coverage across the 15,115 bp mitogenome is shown in blue, with coding sequences (CDS) highlighted as gray boxes and gene names displayed above the coverage plot.
